# Aqueous extract of *Berberis integerrima* root improves renal dysfunction in streptozotocin induced diabetic rats

**Published:** 2013

**Authors:** Hossein Ashraf, Reza Heidari, Vahid Nejati, Minoo Ilkhanipoor

**Affiliations:** 1 *Department of Biology, Faculty of Science, Urmia University, Urmia, I. R. Iran*

**Keywords:** *Berberis integerrima*, Diabetes mellitus, Renal protective, Streptozotocin

## Abstract

**Objective: **Barberry root extract contains various alkaloids that are considered as antioxidants. Beneficial effect of aqueous extract of *Berberis integerrima *root (AEBIR) was evaluated for renal function in diabetic rats induced by STZ.

**Material and Methods:** *Diabetes was induced by i.p. injection of* streptozotocin* (65 mg/kg bw) to rats, after 15 h* *of fasting. Diabetic rats were randomly grouped and treated* daily* with AEBIR and *glibenclamide by gavage *for 42 days.* After 6 weeks of study, all the rats were sacrificed and some biochemical parameters of serum and urine were measured *and* their kidneys tissues were processed for light microscopy.

**Results:** Streptozotocin induced a significant rise in fasting blood glucose, serum creatinine, **blood urea nitrogen**, urine glucose, urine protein, urine albumin, and water intake and a significant decrease in body weight, serum protein, urine urea, and urine creatinine. There was a significant restoration of these parameters to near normal after administration of the AEBIR and also by glibenclamide (0.6 mg/kg bw). The activity of the extract at dose of 500 mg/kg in all parameters except blood glucose and urine glucose was more than that of the standard drug, glibenclamide (0.6 mg/kg, p.o.). Histopathological changes of kidney samples were comparable with respective control.

***Conclusion: ***These results suggested that aqueous extract of *Berberis Integerrima* root improves renal dysfunction in streptozotocin-induced diabetic rats through controlling blood glucose and renal protective effects.

## Introduction

Diabetes is a major degenerative disease in the world today, that is characterized by hyperglycemia, lipoprotein abnormalities, raised basal metabolic rate, defect in reactive oxygen species scavenging enzymes, and altered intermediary metabolism of major food substances (Edem, 2009 ▶). More than 170 million people worldwide suffer from diabetes and this number is likely to become more than double by 2030 (Wild et al., 2004 ▶). In Iran, approximately 2 million adults have been diagnosed with diabetes and about 4.4 million have impaired fasting glucose (Esteghamati et al., 2008 ▶). In people with Type I diabetes, the diabetic nephropathy is the most important cause of death, of whom, 30- 40% eventually develop end-stage renal failure (Giorgino et al., 2004 ▶). Many studies have shown that good metabolic control is beneficial in slowing the progression of nephropathy in diabetes, and if the duration of diabetes is prolonged before reinstitution of normoglycaemia, nephropathy is not easily reversed (Renu et al., 2004 ▶; Floretto et al., 1998 ▶). Notwithstanding much research work, the diabetic kidney damages are increasing rapidly. Patients with diabetes kidney failure undergo either painful dialysis or kidney transplantation (NIDDK, 2007 ▶), which is both costly and harmful. Presently, researches to develop drugs that slow the progression of diabetic kidney damage with fewer side effects are being conducted, however, showing no significant outcome (Levey, 2002 ▶). This has led to increasing exploration of complementary and alternative medicine from natural sources having potent antidiabetic as well as nephroprotective activity with fewer side effects. Traditional medicines and extracts from medicinal plants have been extensively used as alternative medicine for better control and management of diabetes mellitus (Mahalingam and Krishnan, 2008 ▶). 

*Berberis integerrima* belonging to the family berberidacae is an important medicinal shrub. This plant grows in most regions of Iran, especially in northern and northeastern regions of this country. The harvest time is in November. Due to having secondary metabolites such as Berberine, Oxyacanthine, Bermamine, Palmatine, Jateorrhizine, Columbamine, and Berberubine, this plant has many medicinal properties (Arayne et al., 2007 ▶). Various properties are listed for different parts of barberry plant and these properties have been confirmed in various research. In addition to the antioxidant properties of Barberry fruit (Sabir et al., 1978 ▶), varieties of alkaloids are obtained from root and stem bark, which most important of them is Berberine (Ivanovska and Phlipov, 1999 ▶). Based on studies on Barberry root extract and its main alkaloid (Berberine), the following properties are listed: antioxidants (Sabir et al., 1978 ▶), anti-inflammatory effects (Ivanovska and Phlipov, 1999 ▶), hypoglycemia (Yin et al., 2002 ▶), hypolipidemic (Doggrell, 2005 ▶), collecting free radicals, reduction of oxidative stress (Kumar et al., 2008 ▶), etc. As the evidence of earlier studies shows that the whole plant of *Berberis integerrima* possesses flavonoids, alkaloids, and Berberine which are the major chemical constituents responsible for exhibiting antioxidant activity (Gilgun-Sherki et al., 2001 ▶; Ivanovska and Phlipov, 1999 ▶), the present study has been undertaken to evaluate the protective effects of aqueous extract of *Berberis integerrima *root in kidney tissues in streptozotocin-induced diabetic rats.

## Materials and Methods

***Chemicals***

Streptozotocin (STZ) was purchased from Sigma Chemical Co. (St. Louis, MO, USA). All the remaining chemicals were of highest commercially available grade.

***Plant material***

Wild samples of Barberry root (Berberis integrrima) were collected from suburb of Bavanat city (Fars Province, Iran) during November and December 2011 and identified by the Botany Department of Urmia University. A voucher specimen of the plant was deposited in the herbarium of the Faculty of Sciences, Urmia University, Urmia, Iran (No. 9059). 

***Preparation of aqueous extract***

Roots were dried in the shade after washing with cold water and then were powdered using dry grinder and passed through sieve. The aqueous extract was prepared by cold maceration of 150 g of powdered root barks in 500 ml of distilled water for 72 h. Then, the extract was filtered, concentrated, dried in vacuum (yield 10 g) and the residue was stored in a refrigerator at 2-8 °C for use in subsequent experiments (Nawel et al., 2011 ▶).

***Animals***

Male Wistar rats weighing 180-220 g (obtained from the Pasteur Institute central animal house, Tehran, Iran) were housed in an air conditioned room under a 12:12 h light-dark cycle. The animals were allowed free access to tap water and standard laboratory rat food. All experimental procedures involving animals were approved by the Animal Research Ethics Committee of Urmia University of Faculty of Sciences, Urmia, Iran. 

**Acute toxicity study**

*Acute toxicity study of* *aqueous extract of Berberis integerrima root was determined as per the* *OECD guideline No. 423 (Acute Toxic Class Method). It was observed that test extract was not lethal to the rats even at 2500 mg/kg dose. Hence, 10% (250 mg/kg) and 20% (500 mg/kg) of this dose were selected for further study (*Gandhimathi and Sreedevi, 2012 ▶*).*

***Experimental induction of diabetes***

Diabetes was induced in rats by intraperitoneal (i.p.) injection of streptozotocin (STZ) at a dose of 65 mg/kg bw, dissolved in 0.1 cold citrate buffer (pH=4.5) (Sancheti et al., 2010 ▶). Blood samples were taken from the tail vein 72 h after STZ injection to measure blood glucose levels by ACCU-Check glucose meter. Only animals with fasting blood glucose levels (after fasting for 12 h) over 300 mg/dl were considered diabetic and used for the further study (Hosseinzadeh et al., 2002 ▶).

***Experimental design***

All animals were randomly divided into six groups with six animals in each group (Nawel et al., 2011 ▶).

1. Control treated with normal saline (10 ml/ kg).

2. Positive control treated with aqueous extract of Berberis integrrima root (500 mg/kg body weight).

3. Sham treated. Diabetic rats received normal saline (10 ml/kg).

4. Diabetic rats treated with aqueous extract of Berberis integrrima root (250 mg/kg body weight).

5. Diabetic rats treated with aqueous extract of Berberis integrrima root (500 mg/kg body weight).

6. Diabetic rats treated with glibenclamide (0.6 mg/kg of body weight) (Kazemi et al., 2010 ▶).

Animals were treated daily by gavage for 6 consecutive weeks. At the end of the study, the body weight in experimental animals was determined 6 weeks after the study by a digital balance. Animals were accommodated in metabolic cages for urine collection for 2 days in order to become familiar with the environment of the cage. Twenty-four h urine samples were collected from all groups to determine urine parameters. Then, animals were fasted overnight and anesthetized with chloroform (Pharmaceutical Partners of Japan). Blood samples were collected from the animals' hearts and the serum was separated by centrifugation (3000 rpm at 4°C for 15 min) and stored at -20 °C for different biochemical analyses.

***Estimation of*** ***some serum and urine biochemical parameters***

Some biochemical parameters in serum including fasting blood glucose,** blood urea nitrogen,** protein, creatinine, and in urine including urea, albumin, protein, glucose, and creatinine were determined with the use of commercially available enzyme kits (Pars Azmoon, Tehran, Iran) and using an automatic analyzer (Architect c8000 Clinical Chemistry System, USA). 

***Histopathology study***

Kidneys were dissected out and the kidney samples were excised from the experimental animals of each group and washed with the normal saline. The materials were fixed in 10% buffered neutral formalin. They were processed for paraffin embedding following the microtome technique. The sections were taken at 5 μm thickness processed in alcohol‐xylene series and were stained with Periodic Acid Schiff [PAS]. The sections were examined microscopically for the evaluation of histopathological changes.

**Statistical analysis**

All Biochemical data are expressed as mean±SEM. Statistical analysis was performed using one‐way ANOVA followed by Tukey’s multiple tests using SPSS (version 18) of computer software. In all cases, a p-value of less than 0.05 was considered to be significant. 

## Results

**Estimation of** **body weight, serum glucose and water intak** ***e***

Table 1 shows the effect of aqueous extracts of AEBIR and glibenclamide on body weight, serum glucose, and water intake. The levels of glucose in serum of STZ-induced diabetic rats were significantly (p<0.001) elevated when compared with control rats. Administration of AEBIR (250 and 500 mg/ kg bw) or glibenclamide (0.6 mg/kg bw) to diabetic rats for 42 days caused significant reduction (p<0.001) in serum glucose level in comparison with diabetic control. There was a considerable reduction (p<0.001) in the body weight of the diabetic rats as compared with the normal ones. Extract (250 and 500 mg/ kg bw) or glibenclamide (0.6 mg/kg bw) treated groups showed an increase (p<0.001) in body weight in comparison with diabetic control. Moreover, the fluid intake of STZ-induced diabetic rats were significantly (p<0.001) elevated when compared with the control rats. An elevated level of fluid intake by diabetic rats was lowered by the treatment with extracts (250 and 500 mg/ kg bw) and also by the glibenclamide (0.6 mg/kg of bw). 

**Table 1:** Effect of glibenclamide and AEBIR on body weight, serum glucose, and water intake in diabetic rats

** Gorups**	**NC** 10 ml/kg saline	**N+AEBIR** (500 mg/kg bw)	**DC** (10 ml/kg saline)	**D+AEBIR** (250 mg/kg bw)	**D+AEBIR** (500 mg/kg bw)	**D+G** (0.6 mg/kg bw)
**Parameters**						
**FBG (mg/dl)**	93.80±3.35	94.00±2.40	337.20±7.53^a^	162.40±2.61^b^	110.40±.92^b^	113.00±3.43^b^
**Body Weight (g)**	247.62±1.87	236.86±3.18	137.98±2.24^a^	187.56±3.73^b^	200.22±2.78^b^	203.52±5.61^b^
**Water intake (ml/day)**	34.60±2.87	38.80±2.41	172.00±7.06^a^	64.40±2.46^b^	50.00±4.20^b^	64.40±3.35^b^

AEBIR: Aqueous extract of* Berberis integerrima* root, N: normal, C: control, G: glibenclamide, D: diabetic. Values are presented as mean±SEM; n=6 in each group. One way ANOVA followed by Tukey’s test.

a p<0.0001: diabetic controls were compared with normal controls.

b p<0.001 diabetic-treated rats were compared with diabetic control.

**Kidney parameters**

The mean values of serum creatinine, serum protein, blood urea nitrogen, urine urea, urine creatinine, urine albumin, urine glucose, and urine protein, of both control and experimental groups, are presented in Table 2. STZ-induced diabetic rats showed a significant increase (p<0.001) in serum creatinine, blood urea nitrogen, urine albumin, urine glucose, and urine protein and a significant decrease (p<0.001) in urine urea, urine creatinine, and serum protein compared with the normal control. There was a significant restoration of these parameters to near normal after administration of the AEBIR and also by glibenclamide (0.6 mg/kg bw).

**Histopathology of kidney**

**Figure 1:**
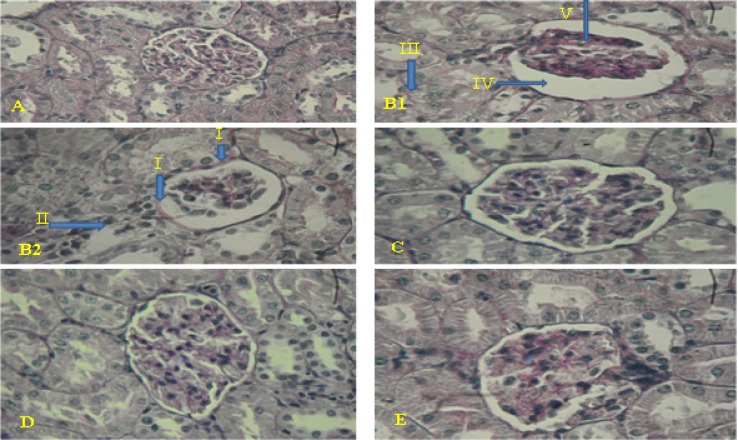
Histopathological evalution of kidney sections. Formalin fixed kidney sections of *5* *μ* thickness from control and diabetic were stained with PAS and images were taken at the magnification of 400×. Various panels represent control kidney (A), diabetic kidney (B1, B2), diabetic kidney treated by 250 mg/kg of AEBIR (C), diabetic kidney treated by 500 mg/kg of AEBIR (D), diabetic kidney treated by 0.6 mg/kg of glibenclamide (E). Mild thickening of the basement membrane (I), mild change density of mesenchyme (II), atrophy of glomerular capillaries (V), increased Bowman's space )urinary space( ( IV) and acute tubular necrosis (III). AEBIR: aqueous extract of *Berberis integerrima *root

Histology of kidney in control animals showed normal structure. In diabetic rats, kidney sections showed mild thickening of the basement membrane along with mild change in the density of mesenchyme, atrophy of glomerular capillaries with increased Bowman's space (urinary space), and acute tubular necrosis (ATN). The groups that were treated with AEBI R (250 and 500mg/ kg bw) or glibenclamide (0.6 mg/kg bw) showed features of healing, i.e., normal glomerulus, normal basement membrane, and capillaries. Moreover, Bowman's space (urinary space) and acute tubular necrosis (ATN) were improved towards normal condition after treatment with AEBI R (250 and 500mg/ kg bw) or glibenclamide (0.6 mg/kg bw) (Figure 1).

**Table 2:** Effects of glibenclamide and AEBIR on kidney parameters in diabetic rats

** Gorups**	**NC** 10 ml/kg saline	**N+AEBIR** (500 mg/kg bw)	**DC** (10 ml/kg saline)	**D+AEBIR** (250 mg/kg bw)	**D+AEBIR** (500 mg/kg bw)	**D+G** (0.6 mg/kg bw)
**Parameters**						
**Serum Creatinine (mg/dl)**	0.66±0.02	0.61±0.00	1.70±0.10^a^	1.00±0.13^c^	.80±0.14^d^	.93±0.06^c^
**Serum Protein (mg/dl)**	2.06±0.08	2.30±0.23	0.49±0.05^a^	1.15±0.05^c^	1.80±0.10^d^	1.12±0.08^b^
Blood urea nitrogen (mg/dl)	9.13±0.58	8.30±0.64	32.54±0.85^a^	14.68±1.14^d^	11.20±0.66^d^	12.27±0.63^d^
**Urine Creatinine (mg/dl)**	67.30±2.33	66.97±2.47	16.36±1.02^a^	26.12±1.55^b^	33.66±2.40^d^	30.20±0.92^d^
**Urine Urea (mg/dl)**	2.74±0.12	2.88±0.11	0.63±0.10^a^	1.70±0.28^b^	2.54±0.22^d^	1.98±0.27^c^
**Urine Proteine (mg/dl)**	17.68±0.89	16.98±0.76	35.91±2.68^a^	22.04±2.09^d^	20.30±0.57^d^	21.34±0.79^d^
**Urine Albumine (mg/dl)**	2.30±0.13	1.18±0.12	23.02±0.98^a^	8.70±0.46^d^	4.51±0.33^d^	7.40±0.22^d^
**Urine glucose (mg/dl)**	13.28±0.41	12.81±0.31	528.60±10.00^a^	198.20±3.51^d^	135.80±5.81^d^	130.80±7.65^d^

AEBIR: Aqueous extract of *Berberis integerrima* root, N: normal, C: control, G: glibenclamide, D: diabetic. Values are presented as mean±SEM; n = 6 in each group. One way ANOVA followed by Tukey’s test,

a p<0.0001: diabetic controls were compared with normal controls.

b p<0.05 and

c p<0.01,

d p<0.001: diabetic treated rats were compared with diabetic control.

## Discussion

The main function of the kidneys is to excrete the waste products of metabolism and to regulate the body concentration of water and salt. STZ administration elevated renal markers, i.e., serum urea nitrogen, creatinine, urine albumin, urine glucose, and urine protein and decreased urine urea and urine creatinine which are found responsible for proper maintenance, functioning of kidney, and change in the glomerular filtration rate (Alderson et al., 2004 ▶; Mauer et al., 1981 ▶). 

These changes were observed in the present study, while AEBI R (250 and 500 mg/ kg bw) or glibenclamide (0.6 mg/kg bw) decreased the level of fasting blood glucose, water intake, serum urea nitrogen, serum creatinine, urine protein, urine albumin, and urine glucose and increased body weight, serum protein, urine urea, and urine creatinine. Hence, our current study confirmed that aqueous extract-treated diabetic rats showed significant improvement in renal functions such as urine protein and urine albumin. It has been reported that in STZ-induced diabetic rats, the renal pathological changes and deteriorated functions are very similar to human diabetes (Stambe et al., 2003 ▶).

Alloxan monohydrate and streptozotocin are drugs that selectively destroy ß-cells of pancreas and thus induce experimental diabetes. Moreover, it has been reported that in Alloxan monohydrate and STZ-induced diabetic rats, the renal and liver undergo pathological changes (Sun et al., 2008 ▶; Stambe et al., 2003 ▶). Glomerular and tubular hypertrophy, increased basement membrane thickness, tubulointerstitial fibrosis, and arteriosclerosis are the pathologic features of diabetic nephropathy. These features are primarily the extent of diffuse mesangial matrix expansion and increased albuminuria that correlate best with progression to renal failure (Haneda, 2006 ▶). 

Many studies have been shown a significant increase in the rate of kidney cell damage (nephropathy) in diabetes disorders (Ahmed and Osman, 2006 ▶; Rashki et al., 2009 ▶; Liu et al., 2008 ▶). Finally, this nephropathy reduces the physiological function and changes in the structure of kidney in diabetes disease (Rosolowsky et al., 2008 ▶). Hyperglycemia increases the generation of free radicals by glucose auto-oxidation and the increment of free radicals may lead to kidney cells damage (Sharma et al., 2006 ▶). 

Presence of flavonoids, alkaloids, glycosides, phenolics, and tannins in the phytochemical screening of the plants are likely to be responsible for the antidiabetic effects (Manickam et al., 1997 ▶; Akowuah et al., 2002 ▶). One of these studies was conducted by Rashki et al., which demonstrated that administration of the garlic Juice (1 ml/100g bw) significantly decreased the serum urea and creatinine and a significantly increased in urine urea, urine creatinine, and creatinine clearance when compared with the control diabetic rats (Rashki et al., 2009 ▶). Moreover, a variety of alkaloids can be obtained from root and stem bark of barberry plant, which Berberine is the most important of (Ivanovska and Phlipov, 1999 ▶). In a study that showed the administration of Berberine (200 mg/kg/d) caused a significantly ameliorated the ratio of kidney weight to body weight, FBG, serum urea nitrogen, creatinine, and 24-h urinary protein were significantly decreased in the Berberine treatment group compared with the diabetic model group (Liu et al., 2008 ▶). In addition, in another study, authors reported that the administration of the Berberine (100 and 200 mg/kg bw) to diabetic rats caused a significant decrease in fasting blood glucose, insulin levels, total cholesterol, triglyceride levels, urinary protein excretion, serum creatinine, and blood urea nitrogen (Wu et al., 2012 ▶) .

Berberine also enhances the hypoglycemic action of insulin in diabetic animal models (Ko et al., 2005 ▶). Recent evidence has demonstrated that Berberine ameliorates proteinuria in type 2 diabetic rats (Si et al., 2005 ▶). Therefore, it is possible that Barberry root extract through their secondary metabolites especially Berberine, is able to reduce blood glucose and protect the kidneys in diabetic patients. 

From the overall results of the biochemical and histopathological examinations, it could be inferred that aqueous extract of *Berberis integerrima* showed the beneficial effect (especially at dose of 500 mg/kg bw) on renal function in STZ-induced diabetic rats. Further study on aqueous extract of *Berberis integerrima* could be extended for the isolation and structure determination of the beneficial effect on renal and liver function principle(s).

## References

[B1] Ahmed MH, Osman MM (2006). Improving laboratory diagnosis of diabetic nephropathy with the use of glomerular filtration rate. Diabetes Technol Ther.

[B2] Akowuah GA, Sadikun A, Mariam A (2002). Flavonoid identifi-cation and hypoglycaemic studies of the butanol fraction from Gynura procumbens. Pharm Biol.

[B3] Alderson NL, Chachich ME, Frizzell N, Canning P, Metz TO et al (2004). Effect of antioxidants and ACE inhibition on chemical modification of proteins and progression of nephropathy in streptozotocin diabetic rat. Diabetologia.

[B4] Arayne MS, Sultana N, Bahadur SS (2007). The berberis story: Berberis vulgaris in therapeutics. Pak J Sci.

[B5] Doggrell SA (2005). Berberine-a novel approach to cholesterol lowering. Expert Opinion Investing Drugs.

[B6] Edem DO (2009). Hypoglycemic effects of ethanolic extracts of Alligator Pear Seed (Persea Americana Mill) in rats. Eur J Sci Res.

[B7] Esteghamati A, Gouya MM, Abbasi M, Delavari A, Alikhani S (2008). Ptevalence of diabetes and impaired fasting glucose in the adult population of iran:National Survey of Risk Faktors for Non Communicable Diseases of Iran. Diabetes-care.

[B8] Floretto P, Steffes MW, Sutherland ERD, Goetz CF, Mauer M (1998). Reversal of lesions of diabetic nephropathy after pancreas transplantation. NEJM.

[B9] Gandhimathi R, Sreedevi A (2012). Cerebro-protective effect of synedrella nodiflora linn. against cerebral ischemia in rats. Int J of Pharmacol Res.

[B10] Gilgun-Sherki Y, Melamed E, Offen D (2001). Oxidative stress induced neuro-dengenerative diseases: The need for antioxidants that penetrate the blood brain barrier. Neuropharmacology.

[B11] Giorgino F, Lavida L, Cavallo PP, Solnica B, Fuller J (2004). Factors associated with progression to macroalbuminuria in microalbuminuria type 1 patients: Eurodiab prospective and complications study. Diabetologia.

[B12] Haneda M (2006). Mechanisms for the development and progression of diabetic nephropathy. Nippon Rinsho.

[B13] Hosseinzadeh H, Ramzani M, Danaei AR (2002). Antihyperglysemic effect and acute toxicity of Securigera securidaca L. seed Extracts in mice. Phyto Res.

[B14] Ivanovska N, Phlipov S (1999). Study on the anti-inflammatory action of berberis vulgaris root extract, alkaloid fractions and pure alkaloid. Int J Ethnopharmacol.

[B15] Kazemi S, Asgary S, Moshtaghian J, Rafieian M, Adelnia A (2010). Liver-protective effects of hydroalcoholic extract of Allium Hirtifolium Boiss in rats with alloxan-induced diabetes mellitus. ARYA Atherosclerosis Journal.

[B16] Ko BS, Choi SB, Park SK, Jang JS, Kim YE (2005). Insulin sensitizing and insulinotropic action of berberine from Cortidis Rhizoma. Biol Pharm Bull.

[B17] Kumar S, Kumar D, Rakash O (2008). Evaluation of antioxidant potential, phenolic and flavonoid contents of hibiscus liliaceous flowers. EJAF.

[B18] Levey AS (2002). Nondiabetic kidney disease. N Engl J Med.

[B19] Li WL, Zheng HC, Bukuru J, De KN (2004). Natural medicines used in the traditional Chinese medical system for therapy of diabetes mellitus. J Ethnopharmacol.

[B20] Liu W, Hei Z, Nie H, Tang F, Huang H (2008). Berberine ameliorates renal injury in streptozotocin-induced diabetic rats by suppression of both oxidative stress and aldose reductase. Chin Med J.

[B21] Mahalingam G, Krishnan K (2008). Anti-diabetic and Ameliorative Potential of Ficus bengalensis Bark extract in Streptozotocin- induced diabetic rats. Ind J of Clin Bioch.

[B22] Manickam M, Ramanathan M, Jahromi MA, Chansouria JP, Ray AB (1997). Antihyperglycaemic activity of phenolics from Pterocarpus marsupium. J Nat Prod.

[B23] Mauer SM, Steffes MW, Brown DM.1981 (The kidney in diabetes). Am J Med.

[B24] Nawel M, Mohamed E, Amine D, Hocine A, Boufeldja T (2011). Hypoglycaemic effect of Berberis vulgaris L. in normal and streptozotocin-induced diabetic rats. Asian Pac J Trop Biomed.

[B25] NIDDK (National Institute of Diabetes and Digestive and Kidney Diseases) (2007). Kidney.

[B26] Rashki KM, Gol A, Dabiri SH (2009). Preventive Effects of Garlic Juice on Renal Damages Induced by Diabetes Mellitus in Rats. Iran J Endocrinol Metab.

[B27] Renu A, Saiyada NA, Odenbach S (2004). Effect of reinstitution of good metabolic control on oxidative stress in kidney of diabetic rats. J Diab Compl.

[B28] Rosolowsky ET, Niewczas MA, Ficociello LH, Perkins BA, Warram JH (2008). Between hyperfiltration and impairment: demystifying early renal functional changes in diabetic nephropathy. Diabetes Res Clin Pract.

[B29] Sabir M, Akhter MH, Bhide NK (1978). Ffurther studies on pharmacology of berberin. India J Physio Pharmacol.

[B30] Sancheti S, Sancheti S, Bafna M, Seo SY (2010). Antihyperglycemic, antihyperlipidemic, and antioxidant effects of Chaenomeles sinensis fruit extract in streptozotocin induced diabetic rats. Eur Food Res Technol.

[B31] Sharma S, Kulkarni SK, Chopra K (2006). Curcumin, the active principle of turmeric (Curcuma longa), ameliorates diabetic nephropathy in rats. Clin Exp Pharmacol Physiol.

[B32] Si FX, Yang DS, Wang BX (2005). Renal protection of berberine and its mechanism in type diabetic rats. Heilongjiang Med Pharmacy (Chin).

[B33] Stambe C, Atkins RC, Tesch GH, Kapoun AM, Hill PA (2003). Blockade of p38alpha MAPK ameliorates acute inflammatory renal injury in rat anti-GBM glomerulonephritis. J Am Soc Nephrol.

[B34] Sun JE, Ao ZH, Lu ZM, Xu HY, Zhang XM (2008). Antihyperglycemic and antilipidperoxidative effects of dry matter of culture broth of Inonotus obliquus in submerged culture on normal and alloxan-diabetes mice. J Ethnopharmacol.

[B35] Wild S, Roglic G, Green A, Sicree R, King H (2004). Global prevalence of diabetes: estimates for the year 2000 and projecttions for 2030. Diabetjutjtes Care.

[B36] Wu D, Wen W, Qi CL, Zhao RX, Lu JH (2012). Ameliorative effect of berberine on renal damage in rats with diabetes induced by high-fat diet and streptozotocin. Phytomedicine.

[B37] Yin I, Hu R, Chen M, Tang J, Li F (2002). Effects of berberine on glucose metabolism in vitro. Metabolism.

